# Genetically proxied tumour necrosis factor inhibition and pregnancy-related maternal and foetal outcomes in the general population: a Mendelian randomization study

**DOI:** 10.1093/rap/rkaf100

**Published:** 2025-08-29

**Authors:** Sizheng Steven Zhao, Benjamin Woolf, Tormod Rogne, Dipender Gill

**Affiliations:** Centre for Musculoskeletal Research, Division of Musculoskeletal and Dermatological Sciences, School of Biological Sciences, Faculty of Biology Medicine and Health, The University of Manchester, Manchester Academic Health Science Centre, Manchester, UK; School of Psychological Science and MRC Integrative Epidemiology Unit, University of Bristol, Bristol, UK; MRC Biostatistics Unit at the University of Cambridge, Cambridge, UK; Department of Chronic Disease Epidemiology, Yale School of Public Health, New Haven, CT, USA; Center for Perinatal, Pediatric and Environmental Epidemiology, Yale School of Public Health, New Haven, CT, USA; Department of Epidemiology and Biostatistics, School of Public Health, Imperial College London, London, UK

**Keywords:** TNF inhibitor, gestational diabetes, preterm birth, spontaneous abortion, birthweight, eclampsia, ectopic pregnancy, hyperemesis gravidarum

## Abstract

**Objectives:**

Evidence on the safety of TNF inhibitors (TNFi) for pregnancy-related maternal and foetal outcomes remains limited. While some studies report increased rates of preterm delivery, others have suggested a possible protective role for gestational diabetes. We used population-level data to examine the effect of genetically proxied TNFi on these outcomes.

**Methods:**

We proxied TNFi using rs1800693, a splicing variant within the *TNFRSF1A* gene, which is strongly associated with CRP in a genome-wide association study of 575 531 individuals of European ancestry. CRP was selected as the biomarker because TNFi is recognized to suppression CRP. Genetic association data for pregnancy-related outcomes were taken from FinnGen and UK Biobank, including the outcomes of spontaneous abortion, ectopic pregnancy, hyperemesis gravidarum, gestational diabetes, pre-eclampsia or eclampsia, preterm birth and offspring birthweight. Colocalization analysis was used to examine genetic confounding.

**Results:**

We found no strong association between genetically proxied TNFi and any adverse pregnancy-related outcome, including spontaneous abortion (odds ratio [OR] 1.07, 95% CI: 0.41, 2.81), preterm birth (OR 0.48, 95% CI 0.14, 1.60), hyperemesis gravidarum (OR 0.20, 95% CI 0.02, 2.57), pre-eclampsia or eclampsia (OR 0.59, 95% CI 0.13, 2.65). Genetically proxied TNFi was associated with lower risk of gestational diabetes (OR 0.16, 95% CI 0.05, 0.52). There was no statistical evidence to suggest genetic confounding through linkage disequilibrium.

**Conclusion:**

This genetic investigation found no evidence linking TNFi to adverse pregnancy-related outcomes. The suggestive association with a reduced risk of gestational diabetes warrants further research and may support its consideration for at-risk pregnant women.

Key messages

- The safety of TNFi in pregnancy remains unclear for many with immune-mediated rheumatic diseases.
- No evidence links TNFi to spontaneous abortion, ectopic pregnancy, pre-eclampsia, preterm birth or birthweight.
- TNFi may lower gestational diabetes risk, but confirmation is needed in future research.

## Introduction

TNF inhibitors (TNFi) have significantly improved the management of various immune-mediated inflammatory diseases, such as rheumatoid arthritis, axial spondyloarthritis, psoriatic disease and juvenile idiopathic arthritis. Many individuals with these conditions are of childbearing age and, while evidence continues to emerge, the safety of TNFi on specific and rarer pregnancy-related outcomes remains uncertain [1, 2]. Although certolizumab pegol does not cross the placenta and is unlikely to affect the fetus directly, its potential impact on maternal outcomes is unclear. Clinical trials of TNFi have typically excluded pregnant women, leading to limited data on their potential effects on maternal and foetal health.

Most TNFi are known to cross the placenta, especially during the third trimester [3], raising concerns about potential immunosuppression in newborns [1]. Data on other foetal outcomes are less conclusive. While many studies report no significant increase in adverse outcomes (e.g. miscarriage, stillbirth, or congenital anomalies [1, 4–8]), some have reported higher rates of preterm delivery [9, 10], which might be attributed to the underlying disease. By contrast, observational evidence suggests a possible protective effect of TNFi on gestational diabetes among women with IBD [11]. Lack of evidence in this area can contribute to maternal anxiety, and suspending treatment may lead to disease flares during pregnancy. Although research has understandably focused on foetal outcomes, there is limited information on the effects of TNFi on maternal health during pregnancy.

Generating robust safety data for TNFi in pregnancy is challenging due to ethical constraints on clinical trials [12]. Most existing evidence comes from accidental exposures, which can be subject to bias. Ongoing clinical trials of TNFi use during pregnancy are unlikely to be sufficiently powered to study many important or rare outcomes, such as ectopic pregnancy. Naturally occurring variations in the genes that encode the protein drug target can provide insights into safety, without exposing pregnant women to these drugs. Furthermore, studying population-level data can help differentiate effects of TNFi from those of uncontrolled disease activity on pregnancy-related outcomes [12]. A splicing variant in *TNFR1* (encoding TNF receptor 1) produces a decoy TNF receptor that acts analogously to the TNFi etanercept [13]. Our aim in this work was to study the effect of genetically proxied TNFi on a range of pregnancy-related outcomes, including spontaneous abortion, ectopic pregnancy, hyperemesis gravidarum, gestational diabetes, pre-eclampsia or eclampsia, preterm birth and offspring birthweight.

## Methods

**Figure 1.**
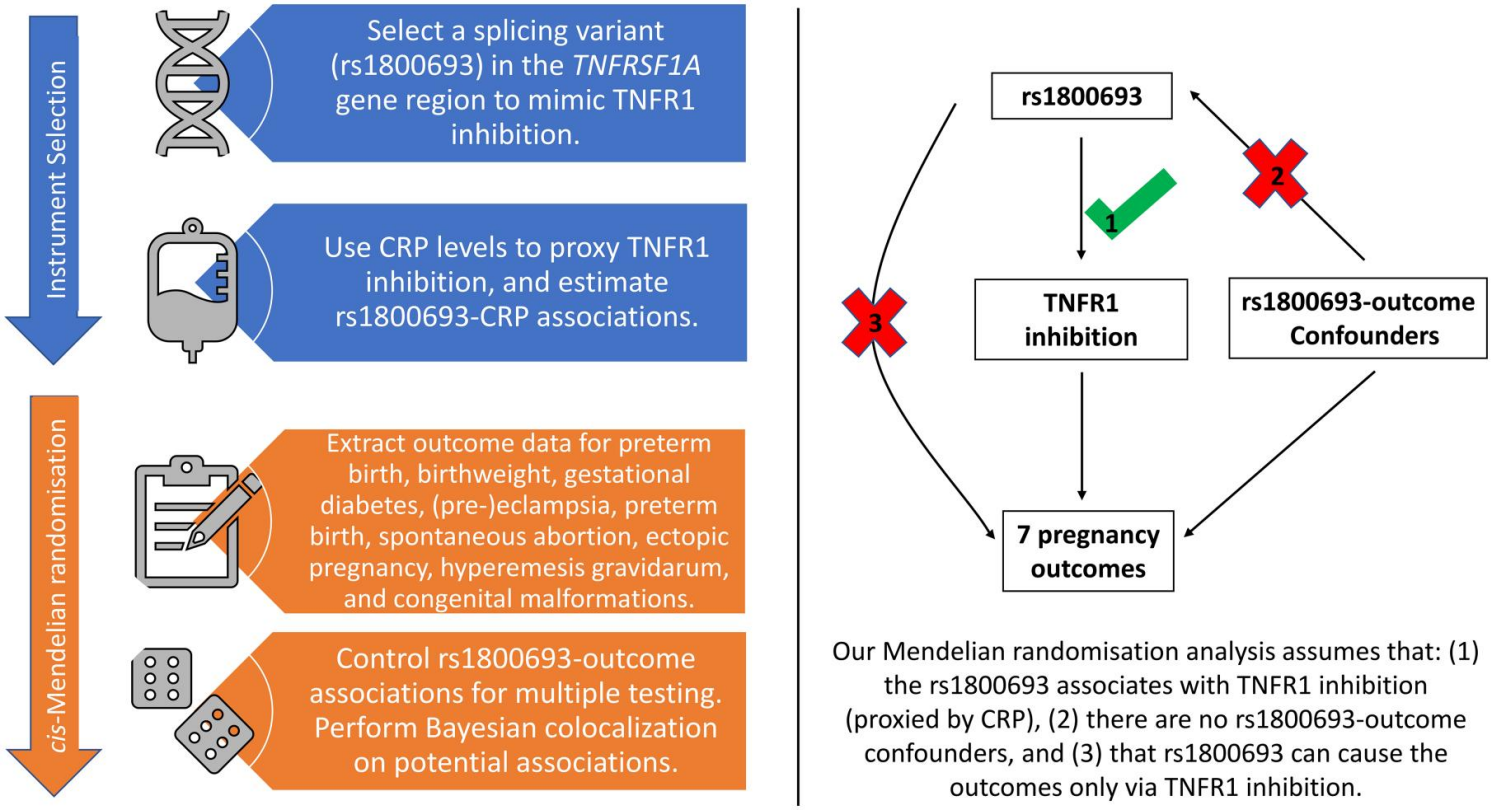
Study overview figure. Outcome data sources are the Early Growth Genetics GWAS Consortium, UK Biobank and FinnGen

This study used publicly available summary-level data from meta-analyses of genome-wide association studies (GWAS) and a two-sample MR design to estimate the effect of TNF inhibition using a functional single-nucleotide polymorphism (SNP) from the *TNFRSF1A* gene as an instrumental variable (Fig. 1). The design can be conceptualized as a general-population-level natural experiment, comparing risk of pregnancy-related outcomes across groups with genetic differences in *TNFRSF1A*. Since genetic variants are randomly inherited during meiosis, differences in this variant are unlikely to be associated with potential confounding traits, thus can be considered analogous to randomization and more robust against confounding than traditional observational designs [14]. This design assesses the biological plausibility of associations with inference that is not derived from a specific IMID population. We used CRP as a biomarker to demonstrate the functional relevance of this variant (i.e. CRP levels differ across groups with different alleles of the *TNFRSF1A* variant) because therapeutic TNFi is well recognized to suppress CRP levels, and GWAS of CRP is much larger and provides greater statistical power than GWAS of circulating TNF levels.

### Data sources

#### C-reactive protein

Genetic association data for CRP were taken from a GWAS meta-analysis of the UK Biobank (UKB) and the Cohorts for Heart and Aging Research in Genomic Epidemiology (CHARGE) consortia comprising 575 531 individuals [15]. Measurements of CRP in the UKB were made using immunoturbidimetry on a Beckman Coulter AU5800. Double genomic control was used to adjust for population structure [16].

#### Spontaneous abortion, ectopic pregnancy, hyperemesis gravidarum, gestational diabetes, pre-eclampsia or eclampsia

Summary statistics for these traits were taken from a meta-analysis of the European sub-sample of the Pan-UKB study (*n* = 420 531) with FinnGen (Release 11) [17]. The FinnGen study is a large (*n* = 453 733) population biobank in Finland, described in detail elsewhere [18, 19]. Cases in FinnGen were identified from medical records, matched with UKB case definitions, and then meta-analysed with comparable GWAS endpoints.

The GWAS meta-analyses included 22 120 individuals who had a spontaneous abortion and 621 630 controls; 7110 with ectopic pregnancy and 623 533 controls; 3201 with hyperemesis gravidarum and 464 049 controls; 16 939 with gestational diabetes and 629 014 controls; 9362 with pre-eclampsia or eclampsia; and 478 554 controls. Both FinnGen and UKB GWASs adjusted for age, sex, the first 10 genetic principal components and genotyping batch [20].

#### Preterm birth and maternal effects on offspring birthweight

Solé-Navais *et al.* [21] meta-analysed GWAS summary data from the Early Growth Genetics GWAS Consortium and the UKB. The European ancestry GWASs used here had 15 419 preterm birth cases (defined via medical records or as a spontaneous delivery <259 days) and 217 881 controls; as well as 210 248 offspring and 101 541 own birthweight measurements. The maternal effects on offspring birthweight GWAS used a weighted linear model to account for the direct genetic effect on offspring birthweight. Each participating study applied SNP quality control procedures and adjusted for principal components of ancestry.

### MR analysis

The *TNFRSF1A* gene (GRCh37/hg19: 12:6437923 to 6451280) encodes TNFR1, the main proinflammatory receptor signalling pathway for TNF [22]. Prior studies have shown that a functional variant, rs1800693, leads to the splicing out of exon 6 of *TNFR1* and loss of the transmembrane domain, with a resulting protein that acts as a soluble decoy receptor analogous to the TNFi etanercept [13]. This genetic proxy for TNF inhibition has been associated with a series of positive controls outcomes, such as ankylosing spondylitis and multiple sclerosis [13, 23, 24].

The primary MR estimator was the Wald ratio, defined as the ratio of the instrument-outcome association to the instrument-CRP association. We harmonized data using the TwoSampleMR R package [25, 26], and used the Benjamini-Hochberg False Discovery Rate (FDR) Procedure to correct for multiple testing.

To examine whether any observed associations are specific to TNF perturbation, or due to CRP reduction in general, we additionally examined the association between CRP and the relevant outcome. We instrumented CRP using independent (r^2^<0.001) variants associated with CRP level at genome-wide significance (*P* < 5 × 10^−8^) in the same CHARGE data. Odds ratios (OR) presented here for binary traits represent the multiplicative increase in the odds for each SD decrease in CRP levels.

### Sensitivity analyses

#### Colocalization

A threat to the validity of single-gene-region MR analyses is confounding by linkage disequilibrium (LD). This occurs when a neighbouring gene in LD with the instrument(s) used in the MR analysis is causal for the outcome of interest. In such settings, genetic instruments can associate with the outcome in the absence of a true exospore-outcome causal effect.

Bayesian colocalization, coloc, is a common sensitivity analysis for confounding by LD [27]. Coloc arbitrates between five hypotheses: H0, that the gene region does not cause either phenotype; H1 and H2, that the gene region causes only one phenotype; H3, that there are separate genetic causes for both phenotypes; and H4, that there is the same genetic cause for both phenotypes. A posterior probability of 80% for H4 is typically regarded as strong evidence for colocalization. However, coloc was developed for GWASs and can therefore be underpowered for detecting associations at sub-genome-wide significance [28]. We implemented coloc for outcomes where we found evidence of an association with genetically predicted TNFR1 blockade using a 75 kb window around rs1800693.

#### Same population assumption

Two-sample MR assumes that the exposure and outcome samples can be treated as being drawn from homogeneous populations. Here we have used a mixed-sex exposure GWAS even though only one sex can become pregnant in order to maximize statistical power. If sex is an effect modifier of the variant-CRP association, then there should be heterogeneity between the summary stats for female only and mixed-sex GWASs. We therefore use the MR SamePopTest sensitivity analysis to test for the presence of heterogeneity between the female-only and mixed-sex CRP GWASs [29].

#### Selection bias

Only females that get pregnant can have negative pregnancy outcomes. As a control for potential collider bias resulting from an effect of TNFi on fertility, we checked if our instrument was associated with female infertility in 16 720 cases and 249 015 controls.

#### Ethics

Ethical approval was obtained in all original studies with relevant citations detailed. No additional ethical approval was required for the current study.

## Results

The rs1800693 variant was associated with CRP levels in the UKB and CHARGE samples (beta = −0.022, SE = 0.002, *P* = 9.55 × 10^−27^). MR SamePopTest did not find evidence to support the presence of heterogeneity between the mixed sex and female-only CRP GWASs (difference in betas = 0.003, se = 0.004, *P* = 0.418). There was also no strong evidence that rs1800693 is associated with female infertility (*P* = 0.429) in FinnGen.

**Figure 2.**
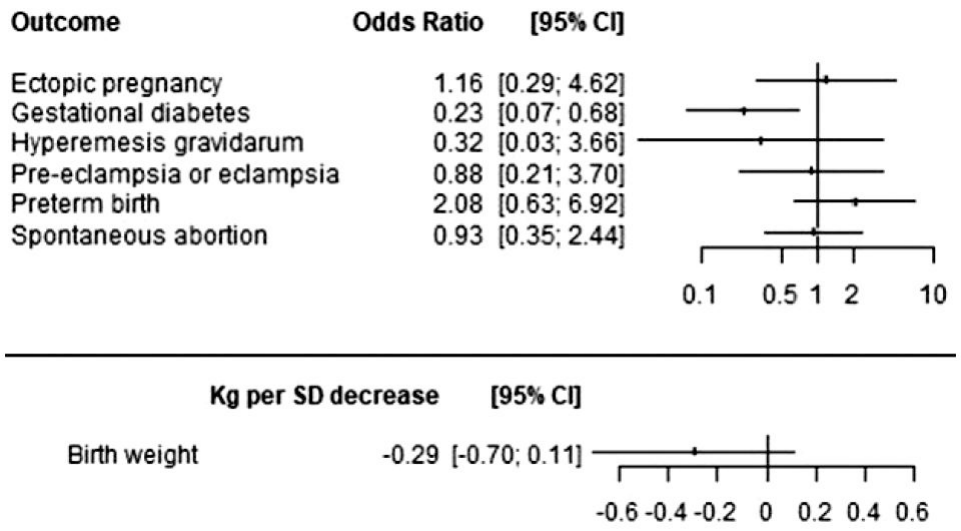
Forest plot of primary MR analyses. ORs represent the multiplicative increase in the odds of the outcome for each SD decrease in CRP levels caused by genetically proxied TNFR1 blockade. Associations with offspring congenital malformation outcomes might represent the direct genetic effect of the variant on the offspring or a pregnancy effect. 95% CI in this figure do not account for multiple testing

Our MR analysis did not find evidence to support an association between genetically proxied TNFi and offspring birthweight (beta = −0.29 kg per SD decrease, 95% CI: −0.70 to 0.11, p_crude_ = 0.153, p_FDR_ = 0.534), ectopic pregnancy (OR = 1.16, 95% CI: 0.29 to 4.62, p_crude_ = 0.833, p_FDR_ = 0.884), hyperemesis gravidarum (OR = 0.23, 95% CI: 0.07 to 3.66, p_crude_ = 0.360, p_FDR_ = 0.630), pre-eclampsia or eclampsia (OR = 0.88, 95% CI: 0.21 to 3.70, p_crude_ = 0.860, p_FDR_ = 0.884), spontaneous abortion (OR = 0.93, 95% CI: 0.35 to 2.44, p_crude_ = 0.232, p_FDR_ = 0.541) and preterm birth (OR = 0.93, 95% CI: 0.35 to 2.44, p_crude_ = 0.884, p_FDR_ = 0.884). However, there was some potential evidence of reduced risk for gestational diabetes, although this did not survive correction for multiple testing (OR = 0.23, 95% CI: 0.07 to 0.68, p_crude_ = 0.008, p_FDR_ = 0.059). Results are summarized in Fig. 2.

**Figure 3.**
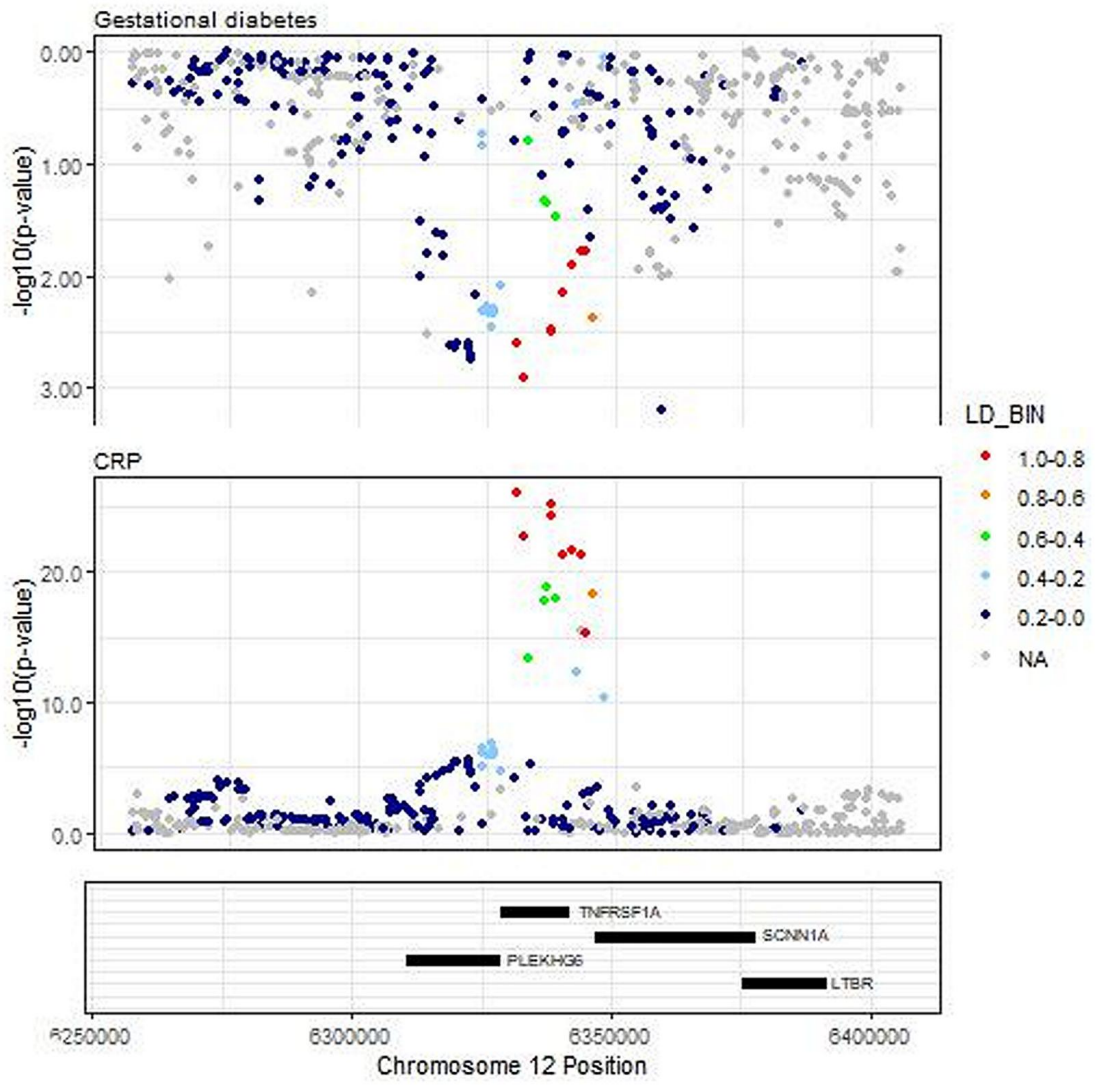
Locus plot for the gene region 75 kb around rs1800693

For the TNFi association with gestational diabetes, there was a larger posterior probability for colocalization than confounding by LD (PP H4 = 36.3% vs PP H3 2.3%), but analysis was limited by low statistical power (Fig. 3). This observed association did not appear to be explained by general reduction in CRP; specifically, genetically predicted CRP level was not associated with gestational diabetes risk (OR 0.94; 95% CI: 0.83 to 1.07).

## Discussion

Evidence on the safety of TNFi during pregnancy is limited and difficult to generate. Using population-level data, our analysis found no evidence that genetically proxied TNFi (using a *TNFR1* variant that is functionally analogous to the action of etanercept) is associated with adverse outcomes. However, we observed suggestive evidence of an association between genetically proxied TNFi and a reduced risk of gestational diabetes, consistent with one prior observational study [11].

The lack of association between TNFi and adverse foetal and maternal outcomes is largely consistent with existing observational evidence [1, 4–8]. The association between TNFi and preterm birth is not consistently reported in the literature [8–10] and was not supported by results from our genetic investigation. Preterm births may be driven by confounding from the underlying inflammatory disease activity. This underscores the strength of using population-level data to examine the biological plausibility of drug effects on outcomes, independent of the underlying disease.

Results from this study provide further evidence against adverse pregnancy-related outcomes associated with TNFi, supporting existing expert opinion-based recommendations that it is safe to continue TNFi throughout pregnancy [1]. Treatment decisions should be made in collaboration with patients, considering all available evidence (including the findings presented here) and the potential for disease flare that might result from suspending TNFi during pregnancy. TNFi that cross the placenta during the third trimester may have implications for childhood vaccinations; therefore, discontinuation in late pregnancy may still be appropriate in certain clinical scenarios.

Gestational diabetes is a common disorder of pregnancy that has substantially increased in prevalence across diverse population groups in the past decade, conferring substantial morbidity to both mother and child [30, 31]. The finding that genetically proxied TNFi might be associated with a lower risk of gestational diabetes is novel and concurs with a recent observational finding that TNFi is associated with lower risk of gestational diabetes among women with IBD [11]. While the analysis did not reach nominal significance after FDR correction, the American Statistics Association has argued against the interpretation of *P*-values based on a dichotomous threshold [32].

There is biological plausibility for this association, as low-grade inflammation is implicated in metabolic syndrome and diabetes [33]. In healthy humans, infusions inducing a relatively minor elevation of TNF inhibit peripheral (predominantly skeletal muscle) insulin-mediated glucose uptake, without influencing endogenous hepatic glucose production [34]. MR studies have provided suggestive evidence that TNF is associated with risk of diabetes [35, 36]. Among non-pregnant IMID populations with type 2 diabetes, observational studies have reported improved glycaemic control after TNFi initiation [37, 38] although interpretation is limited by inherent confounding. However, type 2 diabetes and gestational diabetes may have different genetic underpinnings [39]. To our knowledge, there have been no interventional studies of TNFi on gestational diabetes. The human placenta is an important source of TNF, with the greatest production in late gestation [40] and predominantly secreted into maternal circulation [41]. Placentas from women with gestational diabetes release greater amounts of TNF in response to a glucose stimulus than those from women without [42]. Taken together, there is biological plausibility behind our observed association, but future, better powered studies are needed to confirm whether TNF is implicated in the pathology of gestational diabetes.

There are several important limitations to consider when interpreting our findings. First, our analysis reflects lifelong, subtle, likely cross-tissue perturbation of TNF, which differs from therapeutic TNF inhibition in later life. Second, our results pertain to one population of European ancestry and further replication is required in other populations. Third, as with all MR studies, instrumental variable assumptions are not empirically verifiable. Colocalization analysis was underpowered and genetic confounding (i.e. pleiotropy as a result of variants in linkage with the selected genetic instruments) remains possible, although the posterior probability of a shared causal variant is over 10-fold larger than that of distinct causal variants. Finally, the wide 95% CI in our analyses implies that some outcomes may lack power to detect moderate effects.

In conclusion, we found no evidence to support an association between TNFi and adverse pregnancy outcomes, specifically spontaneous abortion, ectopic pregnancy, hyperemesis gravidarum, pre-eclampsia or eclampsia, preterm birth or offspring birthweight. We show suggestive evidence that TNFi may be associated with reduced risk of gestational diabetes, which should be the focus of future research may support its consideration for at-risk pregnant women.

## Data Availability

UK Biobank data are available to all bona fide researchers for use in health-related research that is in the public interest. The application procedure is described at www.ukbiobank.ac.uk. FinnGen data are available from www.finngen.fi.
